# Nanomechanical Characterization of Plasma-Sprayed Nanostructured Yb_4_Hf_3_O_12_ Thermal/Environmental Barrier Coatings

**DOI:** 10.3390/ma19132875

**Published:** 2026-07-05

**Authors:** Shun Wang, Tao Zheng, Baosheng Xu, Xiaodong Zhang, Yiguang Wang, Feifei Zhou

**Affiliations:** 1 Institute of Advanced Structure Technology, Beijing Institute of Technology, Beijing 100081, China; 2 Marine Science and Technology Domain, Beijing Institute of Technology, Zhuhai 519088, China; 3 Department of Materials Science, School of Materials Science and Engineering, Harbin Institute of Technology, Harbin 150001, China; 4 State Key Laboratory of Advanced Marine Materials, Ningbo Institute of Materials Technology and Engineering, Chinese Academy of Sciences, Ningbo 315201, China

**Keywords:** rare earth hafnate, nanomechanical properties, plasma spraying, nanostructure, thermal/environmental barrier coatings

## Abstract

Thermal/environmental barrier coatings (T/EBCs) have become a notable research field for the development of high-performance thermal protection coatings. The mechanical properties are essential for T/EBCs, which determine the functionality, reliability and durability of coatings. The Yb_4_Hf_3_O_12_ TEBCs were prepared by atmospheric plasma spraying using nanostructured spherical feedstocks and the nanomechanical properties of the Yb_4_Hf_3_O_12_ coatings were characterized by nano-indentation in this work. Results indicate the elastic indentation work (*W_e_*) is 16.06 ± 1.45 nJ and the plastic indentation work is 28.62 ± 6.87 nJ for nanostructured Yb_4_Hf_3_O_12_ coatings. The ratio of plastic work to total deformation work during indentation as the energy dissipation parameter (*η*) is 0.63 ± 0.05 for nanostructured Yb_4_Hf_3_O_12_ coatings and it can be preliminarily inferred that the Yb_4_Hf_3_O_12_ coating may possess favorable erosion resistance, although direct erosion testing is needed for confirmation.

## 1. Introduction

In order to improve the thrust–weight ratio of an aero-engine, the requirement of turbine inlet temperature becomes higher and higher [1,2,3,4]. Silicon carbide fiber-reinforced ceramic matrix composite (SiC_f_/SiC) has both low density and excellent high-temperature mechanical properties [5,6]. It can replace the currently used superalloys as the thermal structure components of aeroengines with high thrust-to-weight ratio, which can effectively increase the turbine front temperature and achieve substantial weight reduction [7,8,9]. The service environment of the new generation of aircraft engine SiC_f_/SiC composite material is more severe, and it is necessary to apply multi-functional thermal/environmental barrier coatings (T/EBCs) onto the surface to ensure the service reliability [10,11,12,13,14,15,16,17].

Recently, rare earth hafnates have been considered as potential surface layer candidates for T/EBCs due to their high-temperature phase stability and low thermal conductivity [18,19,20,21,22]. Wang et al. investigated the thermodynamic properties of Yb_4_Hf_3_O_12_ and observed its notably low thermal conductivity. Additionally, Yb_4_Hf_3_O_12_ exhibited a low thermal expansion coefficient, close to mono-silicate used in EBCs, rendering it suitable for T/EBCs coating systems. Yuan et al. studied the infiltration behavior of natural volcanic ash and synthetic sandy CMAS within a coating at 1250 °C, showing the strong corrosion resistance of the Yb_4_Hf_3_O_12_ coating, particularly against volcanic ash [23]. Most of these studies focus on the thermal and mechanical properties of Yb_4_Hf_3_O_12_ ceramics and the high-temperature chemical properties of coatings, but few studies have deeply studied the mechanical properties of Yb_4_Hf_3_O_12_ coatings, especially the nanomechanical properties.

In this study, the nanostructured Yb_4_Hf_3_O_12_ coating is prepared by atmospheric plasma spraying (APS) using as-prepared nanostructured feedstocks. The microstructure and phase of Yb_4_Hf_3_O_12_ is characterized by scanning electron microscopy (SEM), transmission electron microscopy (TEM), X-ray diffraction (XRD) and Raman spectroscopy (RS). The nanomechanical properties of coatings including indentation work (*W*) and the ratio of plastic work (*W_p_*) to total deformation work (*W_t_*) have been systematically investigated using nano-indentation technology.

## 2. Experiment

### 2.1. Fabrication of Nanostructured Yb_4_Hf_3_O_12_ Coatings

The Yb_4_Hf_3_O_12_ spherical feedstock for atmospheric plasma spraying was prepared by solid phase synthesis of nano-oxide powder followed by a spray drying process. Yb_2_O_3_ and HfO_2_ are both nano-sized materials, with particle diameters ranging from 10 to 40 nanometers. They were provided by Tianjin Detianzhu Non-crystalline Nano Technology Co., Ltd. (Tianjing, China) The Yb_4_Hf_3_O_12_ T/EBCs were prepared by atmospheric plasma spraying (APS). The Yb_4_Hf_3_O_12_ coatings with a thickness of 200 μm were deposited on SiC/Si/mullite/Yb_2_SiO_5_ coatings prepared in our previous work [24]. It should be noted that the spraying power of the Yb_4_Hf_3_O_12_ coating is lower than that of the Yb_2_SiO_5_ environmental barrier coating to ensure its porous structure with lower thermal conductivity. Since the Yb_4_Hf_3_O_12_ coating is sprayed onto the Yb_2_SiO_5_ coating, only the surface of the coating is preheated during the spraying process. The spraying equipment is from Oerlikon Metco Surface Technology Co., Ltd. (Westbury, NY, USA). The specific spraying parameters applied in this work are shown in Table 1.

### 2.2. Characterization of Yb_4_Hf_3_O_12_ Coatings

A scanning electron microscope (SEM, Nova 430, Thermo Fisher Scientific, Waltham, MA, USA) was used to view the cross-section micrograph of the powders and coating. The microstructures of the Yb_4_Hf_3_O_12_ coating were obtained by transmission electron microscopy (TEM, JEM-2100 F, JEOL Ltd., Tokyo, Japan). The InVia™ Confocal Micro-Raman Spectrometer (Renishaw plc, Gloucestershire, UK) was used for the acquisition of the coating Raman signal. The phases of the feed and coating were characterized by X-ray diffraction (Ultima IV, RIGAKU, Tokyo, Japan). The nanomechanical properties of the Yb_4_Hf_3_O_12_ coating were tested on nano-indentation (Anton-Paar, Graz, Austria). In order to ensure the accuracy of the nanomechanical data of the coating, the coating section was polished with 5000-mesh sandpaper first. The nano-indentation experiment was carried out on the polished cross-section of Yb_4_Hf_3_O_12_ coatings at a pressure of 98 mN with a holding time of 10s. In order to ensure the accuracy of the experiment, 30 sample points were taken for each sample, where the 30 indents are divided into 21 molten-region indents and 9 un-melted-region indents.

## 3. Results and Discussion

### 3.1. Microstructure of Yb_4_Hf_3_O_12_ Feedstocks and Corresponding Coatings

**Figure 1:**
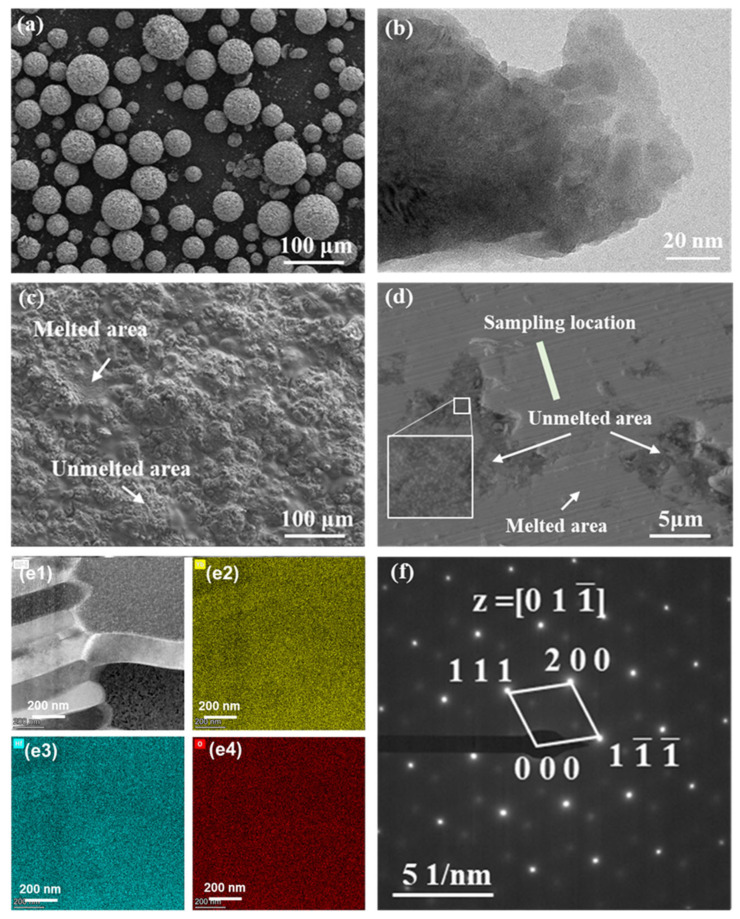
(**a**) Surface morphology of Yb_4_Hf_3_O_12_ feedstocks, (**b**) TEM image of Yb_4_Hf_3_O_12_ feedstocks, (**c**,**d**) surface and cross-section morphology of corresponding coating, (**e1**–**e4**) STEM images of Yb_4_Hf_3_O_12_ coating, and (**f**) SAED pattern results of (**e1**–**e4**).

Figure 1a shows the surface topography of the spherical feedstock of nanostructured Yb_4_Hf_3_O_12_. The results show that the feedstock is spherical with a smooth surface and a particle size of 20~60 μm. The TEM images of the Yb_4_Hf_3_O_12_ feedstocks are shown in Figure 1b. The feedstocks retain their nanostructures during high-temperature sintering. Compared with large particles, small particles are more easily melted during the spraying process. Therefore, the Yb_4_Hf_3_O_12_ coating has the characteristics of a bi-modal structure, as shown in Figure 1c,d. In addition, the coating surface consists of melted lamellae, unmelted and partially melted particles, a certain amount of micropores and a small amount of microcracks. During plasma spraying, the stress caused by the rapid solidification and cooling of the molten particles may be the cause of microcracks. When the high-speed molten particles are deposited on the coating surface, the pores in the coating are inevitable due to the partial filling of the rough surface and the incomplete combination between the particles. This phenomenon is verified by the cross-sectional topography in Figure 1d. Figure 1(e1–e4) is the STEM image of the Yb_4_Hf_3_O_12_ coating, from which it can be seen that there are nanostructures and columnar-like structures in the structure of the coating. Through EDS characterization, we can find that the distribution of Yb, Hf, and O elements was uniform, indicating that the quality of the as-sprayed coating was good. In the diffraction patterns in Figure 1f, it can be found that the phase of the coating is fluorite rather than the common cerium phase. The reason may be that the molten feedstocks cooled rapidly during coating deposition, and the disordered phase did not have time to transform into the ordered phase.

**Figure 2:**
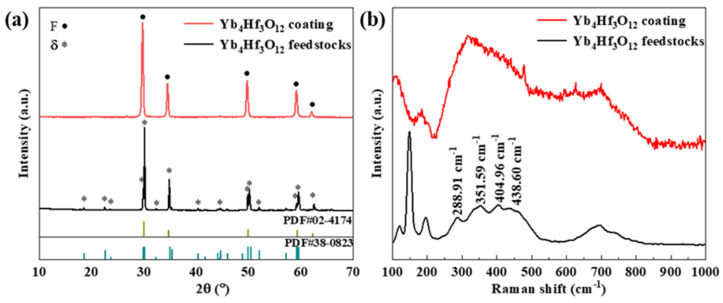
(**a**) XRD and (**b**) Raman spectra of Yb_4_Hf_3_O_12_ feedstocks and coating.

Figure 2a is the XRD pattern of the nanostructured Yb_4_Hf_3_O_12_ coating and feedstock. For the nanostructured Yb_4_Hf_3_O_12_ feedstock, the phase structure is a single phase of δ. After plasma spraying, the phases of the nanostructured Yb_4_Hf_3_O_12_ coating are a fluorite phase and a δ phase. The existence of the fluorite phase in the coating has been analyzed in detail in the previous microstructure characterization of the nanostructured Yb_4_Hf_3_O_12_ coating. For the Yb_4_Hf_3_O_12_-based material, the phase structure includes an ordered δ phase and a disordered pyrochlore phase, namely a defect fluorite phase (F phase). The δ phase and F phase can be distinguished by the presence or absence of (110), (104) and (113) diffraction peaks in the XRD pattern. However, considering that these two diffraction peaks are sometimes very weak, it is not easy to observe and distinguish. Raman spectroscopy is an effective means to characterize the slight changes in crystal structure. The ordered atomic arrangement can be effectively reflected in the Raman scattering, because the corresponding spectral line shape is stronger and clearer. Therefore, the δ phase and F phase can be visually distinguished by the Raman spectroscopy, depending on whether the Raman band is sharp. In order to further confirm the F phase of the nanostructured Yb_4_Hf_3_O_12_ coating, the Raman spectroscopy analysis was carried out, and the results are shown in Figure 2b. Compared with the many sharp Raman bands observed in the feed, the characteristic peaks of the coatings disappeared in the Raman spectra of Yb_4_Hf_3_O_12_-sprayed coatings, and there was a broad peak at about 330 cm^−1^, which belonged to the F phase vibration mode. The broadening of the peak further indicated the disorder of the crystal structure of the coatings, proving that the phase structure of the coatings was F phase. This ordered–disordered transition was mainly due to the rapid cooling of the molten particles during the spraying process, which inhibited the ordered process dynamically [25]. After heat treatment or thermal cycling, the sprayed coatings with F phase can be transformed from the defective F phase to the ordered δ phase. The disordered_ordered transition has no adverse effect on the life of the coatings [23].

### 3.2. Mechanical Properties of Nanostructured Yb_4_Hf_3_O_12_ Coatings

Ceramic coatings face a complex environment of gas and foreign particle erosion during high-temperature service, imposing stringent requirements on their wear resistance [26]. The wear resistance of coatings is largely determined by the accumulation energy on the surface and the dissipative energy during deformation. Nanoindentation testing is a commonly used method to analyze the mechanical properties of plasma-sprayed coatings. However, most studies focus on the results of nano-hardness and elastic modulus, with little in-depth analysis of the load–displacement curves of the coatings, especially the indentation work. The load–displacement curve of nanoindentation mainly consists of three processes: loading, holding load, and unloading, involving the elastic and plastic deformation of the coating and its wear resistance.

**Figure 3:**
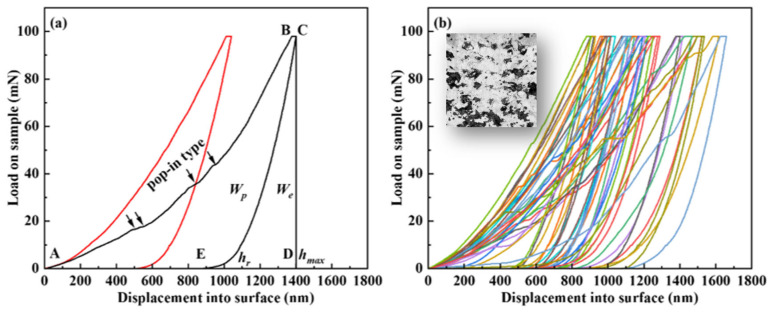
(**a**) Typical load–displacement curves and (**b**) load-displacement curves of Yb_4_Hf_3_O_12_ coatings.

As shown in Figure 3, the load–displacement curves during nanoindentation testing of Yb_4_Hf_3_O_12_ coatings are presented. For ease of analysis, only two typical curves are shown in Figure 3a. The red and black lines represent two characteristic regions of the coating under nanoindentation, namely the molten region and the region with nanostructures present but not melted. The red indentation curve mainly includes smooth loading, holding load, and smooth unloading processes, indicating loading on the molten region of the coating based on our previous studies [27]. However, a weak pop-in phenomenon appears during the loading process of the black indentation load–displacement curve. To quantitatively assess the bimodal nanomechanical response, all 30 nanoindentation test points were examined by SEM after indentation to classify each indent as belonging to either a molten region or an un-melted nano-structured region. Based on this post hoc classification, 21 indents (70%) were located in molten regions, while 9 indents (30%) were located in un-melted regions. This fraction is consistent with the microstructural observation shown in Figure 3b. Combined with the analysis of the microstructure, such curves may represent loading in the insufficiently melted nano-regions of the coating. The occurrence of pop-in-type phenomena may be due to the presence of numerous grain boundaries in the nano-region and weaker bonding between grains in the unmelted region compared to the molten region, resulting in interfacial plasticity between grains under indentation load, making slip between grain boundaries relatively easy. As shown in Figure 3b, the indentation depth of nanoindentations loaded in the molten region is generally smaller than that in the unmelted region, and the indentation depth in the unmelted region has a certain dispersion, which is related to the large differences in microstructure in the unmelted region. The nanomechanical properties exhibit a bi-modal distribution similar to the microstructure.

To further evaluate the wear resistance of the coating, the ratio of plastic indentation work to total indentation work, namely the plastic work ratio (*η*), can characterize the elastoplastic behavior of the coating material [28,29]. As shown in Figure 3a, elastic work can be represented by the area enclosed by the unloading curve and the ordinate (area ECDE), and plastic work can be represented by the area enclosed by the loading–unloading curve (area ABCEA). The total work of indentation can be shown through integral Equation (1):

(1) $$W_{t}=\int_{0}^{h_{max}}P_{load}\left(h\right)dh$$

where *W_t_* represents the total indentation work, *P*_load_(*h*) is the load–displacement function during the loading, and *h_max_* is the maximum depth of indentation. The elastic indentation work can be calculated by Equation (2):

(2) $$W_{e}=\int_{h_{r}}^{h_{max}}P_{unload}\left(h\right)dh$$

where *W_e_* is the elastic indentation work, *P*_unload_(*h*) is the load–displacement function during the unloading and *h_r_* is the residual indentation depth. The calculation of plastic indentation work can be expressed by Equation (3):

(3) $$W_{p}=W_{t}-W_{e}$$

where *W_p_* is the plastic indentation work. The elastic–plastic behavior of coating materials can be characterized by calculating the ratio of the *W_p_* to the *W_t_*, namely the plastic work ratio (*η*), which can reflect the wear resistance of coating materials [28]. The calculation of the *η* can be represented by Equation (4):

(4) $$\eta =\frac{W_{p}}{W_{t}}$$

Through the load–displacement curves of the coating nanoindentation and in conjunction with Equations (1)–(4), the indentation work and *η* value of the bi-modal Yb_4_Hf_3_O_12_ coating can be extracted, thereby providing indirect information for evaluating the potential wear resistance of the coating material.

**Figure 4:**
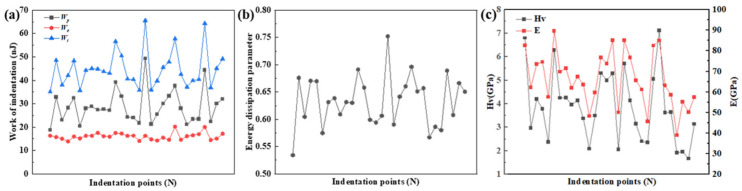
(**a**) Indentation work, (**b**) the η value of Yb_4_Hf_3_O_12_ coatings and (**c**) the nano-hardness (H) and elastic modulus (E) of the Yb_4_Hf_3_O_12_ coating.

As depicted in Figure 4a, the indentation work of the coating is calculated and statistically analyzed. For the Yb_4_Hf_3_O_12_ coating, its elastic indentation work is 16.06 ± 1.45 nJ, while its plastic indentation work is 28.62 ± 6.87 nJ. As mentioned earlier, elastic indentation work reflects energy accumulation, while plastic indentation work reflects energy dissipation [30]. The results indicate that the statistical variance of elastic indentation work for the Yb_4_Hf_3_O_12_ coating is relatively small, whereas the statistical variance of plastic indentation work is larger, suggesting that elastic indentation work is less sensitive to microstructure, while plastic indentation work is more sensitive to microstructure.

Combining the microstructure of the Yb_4_Hf_3_O_12_ coating and the indentation curves, it is evident that the behavior similar to pop-in observed on the nanoindentation curves in the un-melted region dissipates the indentation work to some extent under nanoindentation loading, thereby increasing the coating’s ability to dissipate indentation work [31,32]. Therefore, bi-modal ceramic coatings are expected to prolong the transition time from low-speed wear to rapid wear of the coating. Consequently, it can be tentatively suggested that the Yb_4_Hf_3_O_12_ coating may exhibit promising wear resistance, subject to validation by direct tribological testing.

In addition to indentation work analysis, the nano-hardness (*H*) and elastic modulus (*E*) of the Yb_4_Hf_3_O_12_ coating were also extracted from the nano-indentation load–displacement curves using the Oliver–Pharr method. The average nano-hardness of the coating was determined to be 3.79 ± 1.56 GPa, and the average elastic modulus was 66.60 ± 13.48 GPa. Similar to the plastic indentation work, both hardness and elastic modulus exhibited a bimodal distribution corresponding to the molten and un-melted regions. In the fully molten regions, the hardness reached 4.65 ± 0.96 GPa with an elastic modulus of 73.23 ± 9.34 GPa, while in the un-melted nano-structured regions, lower values of 2.20 ± 0.37 GPa and 52.14 ± 7.77 GPa were observed, respectively. This difference is attributed to the higher porosity and weaker inter-granular bonding in the un-melted regions.

Statistical analysis of the load–displacement curves from these two populations reveals distinct mechanical behaviors. The molten region exhibits higher hardness (4.65 ± 0.96 GPa vs. 2.20 ± 0.37 GPa,) and elastic modulus (73.23 ± 9.34 GPa vs. 52.14 ± 7.77 GPa), while the un-melted region shows higher plastic indentation work (35.72 ± 8.19 nJ vs. 25.54 ± 3.78 nJ) and consequently a higher η value (0.66 ± 0.04 vs. 0.60 ± 0.03). The coefficient of variation (CV) for plastic indentation work is 0.23 for the un-melted region compared to 0.15 for the molten region, indicating greater dispersion in the un-melted region due to microstructural heterogeneity. These statistical results confirm the bimodal nanomechanical response of the coating.

To enable a meaningful comparison with our previous work on 8YSZ thermal barrier coatings [27], Table 2 summarizes the key experimental parameters and nanomechanical results for both studies. It should be noted that the comparison is made under identical nanoindentation conditions (maximum load of 98 mN, loading/unloading rate of 196 mN/min, holding time of 10 s, Berkovich indenter tip) and similar coating architecture (APS-deposited). Under these comparable conditions, the Yb_4_Hf_3_O_12_ coating exhibits approximately 40% higher elastic and plastic indentation work than that of nanostructured 8YSZ coating (16.06 vs. 11.95 nJ for W_e_; 28.62 vs. 20.27 nJ for W_p_), while the *η* value is slightly higher (0.63 vs. 0.62).

## 4. Conclusions

In this paper, the nanomechanical properties of the bi-modal Yb_4_Hf_3_O_12_ coatings prepared by APS were characterized using a nano-indenter. The elastic and plastic indentation work of the Yb_4_Hf_3_O_12_ coating was investigated to calculate the energy dissipation parameters of the coating (*η*). The elastic indentation work is not sensitive to the microstructure, but the plastic indentation work is sensitive to the microstructure, especially to the unmelted region. Based on the value of *η*, it can be indirectly inferred that the bi-modal structure may contribute to enhanced wear resistance, although direct experimental verification is required. It should be noted that these predictions based on indentation work require future validation through dedicated wear, erosion, or tribological measurements under relevant service conditions.

## Figures and Tables

**Table 1:** Atmospheric plasma spraying preparation parameters of Yb_4_Hf_3_O_12_ coating.

APS Layer	Power (kW)	Primary Ar (L/min)	Secondary H_2_ (L/min)	Spray Distance (mm)	Feeding Rate (g/min)
Yb_4_Hf_3_O_12_	44	38	12	120	20

**Table 2:** Experimental and nanomechanical results of Yb_4_Hf_3_O_12_ and 8YSZ.

Parameter	Yb_4_Hf_3_O_12_ (This Work)	8YSZ [27]
Max load (mN)	98	98
Loading/unloading rate (mN/min)	196	196
Holding time (s)	10	10
Indenter type	Berkovich (diamond)	Berkovich (diamond)
W_e_ (nJ)	16.06 ± 1.45	11.95 ± 1.24
W_p_ (nJ)	28.62 ± 6.87	20.27 ± 5.59
*η*	0.63 ± 0.05	0.62 ± 0.05

## Data Availability

The original contributions presented in this study are included in the article. Further inquiries can be directed to the corresponding authors.
